# Recovery-Oriented Mental Health Care: Aligning Lithuanian Mental Health Services with WHO Recovery Principles

**DOI:** 10.1192/j.eurpsy.2026.11257

**Published:** 2026-07-31

**Authors:** A. Germanavičius, G. Skabeikytė Norkienė

**Affiliations:** 1 Republican Vilnius psychiatric hospital; 2 Clinic of Psychiatry, Faculty of Medicine; 3 Instutute of Psychology, Faculty of Philosophy, Vilnius university, Vilnius, Lithuania

## Abstract

**Introduction:**

The World Health Organization defines recovery as a personal, holistic, and human rights-based process that goes beyond the reduction of symptoms. It emphasizes empowerment, autonomy, social inclusion, and meaningful participation in community life. According to WHO guidelines, recovery-oriented care requires sustained support after inpatient treatment to ensure continuity and long-term well-being. Understanding the current gaps and strengths in service provision is essential for aligning psychiatric care with these principles, as since 2019 Lithuania started to implement WHO Quality Rights standards.

**Objectives:**

This presentation aims to provide a semi-theoretical discussion of recovery in mental health, framed by WHO guidelines, and to present an overview of the current situation in Lithuania regarding access to and the actual continuity of outpatient services following inpatient psychiatric hospitalisation as well as Quality Rights standards.

**Methods:**

The analysis combines WHO’s theoretical framework on recovery with national-level data and descriptive information on mental health service utilisation in Lithuania. Particular attention was given to continuity of care after hospital treatment, rehospitalization rates, and accessibility of community-based mental health services.

**Results:**

Preliminary findings indicate that while inpatient psychiatric treatment remains a central element of mental health care in Lithuania, continuity of care through outpatient follow-up is often limited. Despite 115 mental health centres located at primary care level, many patients face challenges in receiving timely and consistent outpatient support, including continuous mental health care and psychosocial rehabilitation, while outpatient crisis intervention and assertive treatment services are yet to be developed. This gap can place recovery at risk, as gains achieved during hospitalisation may not be sustained without adequate follow-up. **Discussion:** the WHO framework highlights the necessity of continuity and person-centered planning to support recovery. In Lithuania, the gap between inpatient treatment and community-based outpatient care undermines the full realization of recovery principles. High rates of rehospitalisation are closely linked to the limited accesibility of crisis intervention outpatient services.

**Conclusions:**

In Lithuania, improving continuity of care through accessible, person-centered outpatient services is essential to implementing WHO’s recovery-oriented approach. This requires government-level decisions to prioritize mental health care, initiate a national program focused on outpatient follow-up and crisis intervention, and ensure adequate financial and human resources. Key priorities include expanding follow-up services, ensuring equitable regional access, integrating psychosocial support, and developing effective outpatient crisis intervention services.

**Disclosure of Interest:**

None Declared

